# Impact and Costs of Incentives to Reduce Attrition in Online Trials: Two Randomized Controlled Trials

**DOI:** 10.2196/jmir.1523

**Published:** 2011-03-02

**Authors:** Zarnie Khadjesari, Elizabeth Murray, Eleftheria Kalaitzaki, Ian R White, Jim McCambridge, Simon G Thompson, Paul Wallace, Christine Godfrey

**Affiliations:** ^5^Department of Health Sciences and HYMSUniversity of YorkYorkUnited Kingdom; ^4^Centre for Research on Drugs and Health BehaviourLondon School of Hygiene & Tropical MedicineLondonUnited Kingdom; ^3^MRC Biostatistics UnitInstitute of Public HealthCambridgeUnited Kingdom; ^2^MRC General Practice Research FrameworkLondonUnited Kingdom; ^1^E-health UnitResearch Department of Primary Care and Population HealthUniversity College LondonLondonUnited Kingdom

**Keywords:** Nonresponse, attrition, Internet, alcohol drinking, randomized controlled trial

## Abstract

**Background:**

Attrition from follow-up is a major methodological challenge in randomized trials. Incentives are known to improve response rates in cross-sectional postal and online surveys, yet few studies have investigated whether they can reduce attrition from follow-up in online trials, which are particularly vulnerable to low follow-up rates.

**Objectives:**

Our objective was to determine the impact of incentives on follow-up rates in an online trial.

**Methods:**

Two randomized controlled trials were embedded in a large online trial of a Web-based intervention to reduce alcohol consumption (the Down Your Drink randomized controlled trial, DYD-RCT). Participants were those in the DYD pilot trial eligible for 3-month follow-up (study 1) and those eligible for 12-month follow-up in the DYD main trial (study 2). Participants in both studies were randomly allocated to receive an offer of an incentive or to receive no offer of an incentive. In study 1, participants in the incentive arm were randomly offered a £5 Amazon.co.uk gift voucher, a £5 charity donation to Cancer Research UK, or entry in a prize draw for £250. In study 2, participants in the incentive arm were offered a £10 Amazon.co.uk gift voucher. The primary outcome was the proportion of participants who completed follow-up questionnaires in the incentive arm(s) compared with the no incentive arm.

**Results:**

In study 1 (n = 1226), there was no significant difference in response rates between those participants offered an incentive (175/615, 29%) and those with no offer (162/611, 27%) (difference = 2%, 95% confidence interval [CI] –3% to 7%). There was no significant difference in response rates among the three different incentives offered. In study 2 (n = 2591), response rates were 9% higher in the group offered an incentive (476/1296, 37%) than in the group not offered an incentive (364/1295, 28%) (difference = 9%, 95% CI 5% to 12%, *P* < .001). The incremental cost per extra successful follow-up in the incentive arm was £110 in study 1 and £52 in study 2.

**Conclusion:**

Whereas an offer of a £10 Amazon.co.uk gift voucher can increase follow-up rates in online trials, an offer of a lower incentive may not. The marginal costs involved require careful consideration.

**Trial registration:**

ISRCTN31070347; http://www.controlled-trials.com/ISRCTN31070347 (Archived by WebCite at http://www.webcitation.org/5wgr5pl3s)

## Introduction

Attrition from follow-up is a major methodological challenge in randomized trials, and the proportion of participants who provide follow-up data is a recognized quality marker [1,2]. Poor follow-up rates reduce the power of analyses and may introduce nonresponse bias, where the likelihood of providing follow-up data is related to the outcome under study [3]. The Internet is increasingly important in the delivery of health care and its evaluation, yet online trials appear to be particularly vulnerable to high rates of attrition from follow-up [4]. Response rates as low as 11% and 15% have been reported at the 3-month follow-up in studies of Web-based health promotion interventions [5,6]. Reasons for the high attrition rates in online trials are unknown. There could be a variety of explanations, such as the ease of entering and leaving an online trial in comparison with a conventional “offline” trial, having little or no direct contact with the research team, or through limited usage or nonusage of the intervention [4].

One approach to increasing response is the use of incentives, which has been effective at increasing response rates in surveys [7-9]. Incentives (such as gift vouchers or lottery participation) have been found to almost double the odds of response to electronic surveys [7]. Varying the levels of incentives was not found to influence response to electronic surveys, although low level comparisons were generally made (eg, US $1 vs US $2) [7]. It is not clear whether these data on improving response in cross-sectional surveys generalize to boosting follow-up in online trials as there are relatively few studies examining this question. One trial of a Web-based program designed to promote healthy eating evaluated 24 different combinations of levels and conditionality of monetary incentives to promote recruitment and retention [10]. The optimal incentive combination was a US $2 unconditional incentive for enrollment and promise of US $20 (conditional incentive) on completion of follow-up measures. The highest rate of retention was achieved with the highest value of incentive. This study thus incentivized recruitment in addition to retention.

With the paucity of empirical research in this area, there is a clear need to evaluate the impact of different incentive levels and types before using them to boost retention in online trials. Even relatively small incentives such as £5 can have an important impact on research budgets, particularly in online trials where large numbers of participants can be recruited reasonably easily [11]. To determine the impact of incentives on follow-up rates in an online randomized trial, we undertook 2 sequential substudies. Both were embedded in a large trial of an online intervention to help hazardous drinkers reduce their alcohol consumption [12,13]. This large study included a pilot phase, followed by the main Down Your Drink trial (the DYD-RCT). Follow-up rates had been identified as an important methodological challenge early in the piloting phase, and a number of initiatives to improve response were tried, including reducing measurement burden by randomizing participants to 1 of 4 secondary outcome measures and adding postal or telephone follow-up to email reminders [14,15]. Despite these attempts, 5 months into the pilot our response rates were low. At this point we decided to explore the use of incentives.

The primary hypothesis in both incentive studies was that offer of an incentive would increase the response rate compared with no offer of incentive. Secondary objectives were to determine the relative effectiveness of 3 different types of incentive (study 1 only), identify predictors of response to incentives, and calculate the cost of achieving an additional response.

## Methods

### Design

We conducted two randomized controlled trials. Ethical approval was obtained for both trials from University College London ethics committee.

### Setting

Both incentive studies were embedded in the Down Your Drink online trial of a Web-based intervention to reduce alcohol consumption [12,13]. The DYD trial and both incentive studies were conducted entirely online (see Textbox 1 for further information on the DYD trial).

### Participants

In both incentive studies, participants were already enrolled in the larger DYD study and, thus, were drinking above recommended levels of alcohol and were interested in reducing their drinking (see Textbox 1). The first incentive study was undertaken with participants in the DYD pilot who did not respond to an email invitation to provide follow-up data within 1 week at its final (3-month) follow-up point. The second study was undertaken with all participants in the main DYD trial at its final (12-month) follow-up point during a defined time period of approximately 9 months.

**Down Your Drink Randomized Controlled Trial**

**Aim**

To determine the effectiveness and cost-effectiveness of the Down Your Drink (DYD) website in reducing alcohol consumption.

**Design**

A 2-arm randomized controlled trial. Participants were randomized to receive access to either an online behavior change program to help people reduce their alcohol consumption or an information-only website on the potential harms of alcohol.

**Methods**

The trial was conducted entirely online through the DYD website [12]. Participants were adults who self-recruited to the trial while looking on the Web for help to reduce their drinking. Visitors to the site were asked to complete a screening test, the 3-item Alcohol Use Disorders Identification Test (AUDIT-C) [16]. Those scoring 5 or more on the AUDIT-C test were invited to participate in the trial. Participants completed baseline measures online before being randomized to 1 of 2 different areas of the website. The intervention area consisted of an extensive behavior change program based on the principles of motivational interviewing, cognitive behavior therapy, behavioral self-control, and relapse prevention [13]. The comparator area of the website consisted of text-based information on the harms of excessive alcohol consumption.

The primary outcome was total past week alcohol consumption, measured by the TOT-AL [17]. Secondary outcomes were: EQ-5D [18], Alcohol Use Disorders Identification Test (AUDIT) [19], Alcohol Problems Questionnaire (APQ) [20], Leeds Dependence Questionnaire (LDQ) [21] and the Clinical Outcomes for Routine Evaluation (CORE-10) measure of mental health [22]. All participants were followed up by email prompt at 1 and 3 months (pilot phase) and 3 and 12 months (main phase).

**Participant profile**

The trial randomized 7935 people who had self-recruited to the trial. The trial recruited slightly more women than men (57%). The majority of participants were white British (84%), with a mean age of 38 years. Around half of the participants were educated to degree level and above (52%). Average alcohol consumption (geometric mean) was 46 (SD 31.2) units per week, where 1 UK unit = 8 g ethanol. Follow-up rates were 55% at one month and 42% at 3 months (pilot trial) and 46% at 3 months and 34% at 12 months (main trial).

### Intervention

#### Study 1

In view of the paucity of literature on incentives for this population, we undertook some preliminary research to identify a range of potentially effective incentives. This included identification of commonly used incentives in the survey literature, discussion with the DYD user representatives, and interviews with a convenience sample of hazardous drinkers demographically similar to the target audience. This preliminary work resulted in the choice of 3 incentives for initial study. Amazon is one of the most popular websites in the United Kingdom, with online shopping being a common use of the Internet [23]. Charitable donations have been widely used in the survey literature [7], with Cancer Research UK being Britain’s largest fundraising charity [24]. We also included an online prize draw (another widely used incentive), which was likely to cost less overall if found to be effective. In light of the current literature, we decided to fix the value of the incentives at £5 (€6 or US $8) for the Amazon voucher and charitable donation and at £250 (€289 or US $387) for the prize draw.

#### Study 2

The results of study 1 informed the decision on level and type of incentive in study 2, for which a £10 Amazon.co.uk voucher was chosen.

In both studies, offer of an incentive was compared with no offer of incentive.

### Study Procedures

#### Study 1

**Figure 1:**
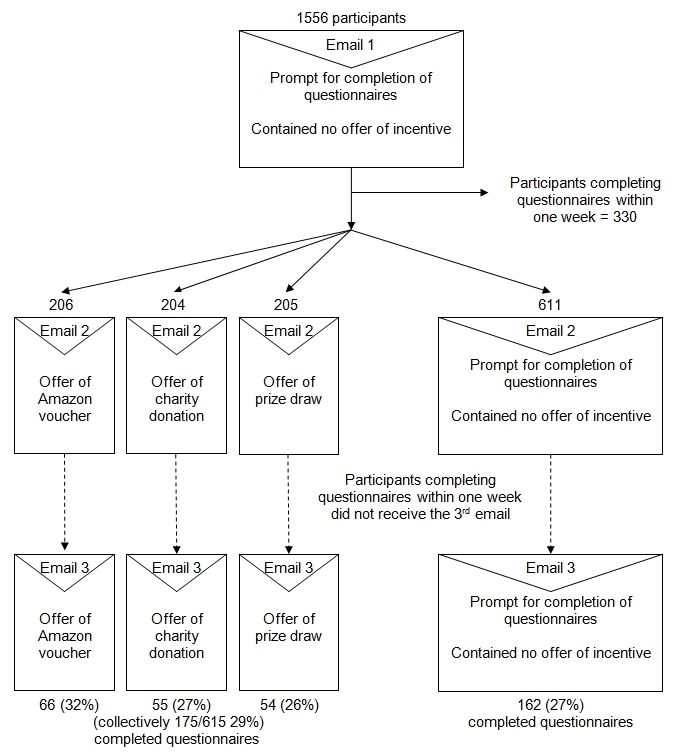
Consolidated Standards of Reporting Trials (CONSORT) flowchart: study 1

In study 1, DYD pilot trial participants were emailed a request to provide follow-up data at 3 months (between September 9, 2007, and January 15, 2008). The email contained a hyperlink to the study questionnaires, stressed the importance of providing follow-up data, and conveyed our gratitude to participants for providing this information. Those participants who had not completed the outcome measures 1 week after the first email request were randomized to receiving an offer of an incentive or no offer of an incentive. Study 1 is thus restricted to those who did not respond to the initial request to provide follow-up data. Within the incentive arm, participants were also randomly allocated to receive either the £5 Amazon.co.uk voucher, £5 donation to Cancer Research UK, or entry in a £250 prize draw. Offer of an incentive was given in the second and third email prompts (Figure 1).

**Figure 2:**
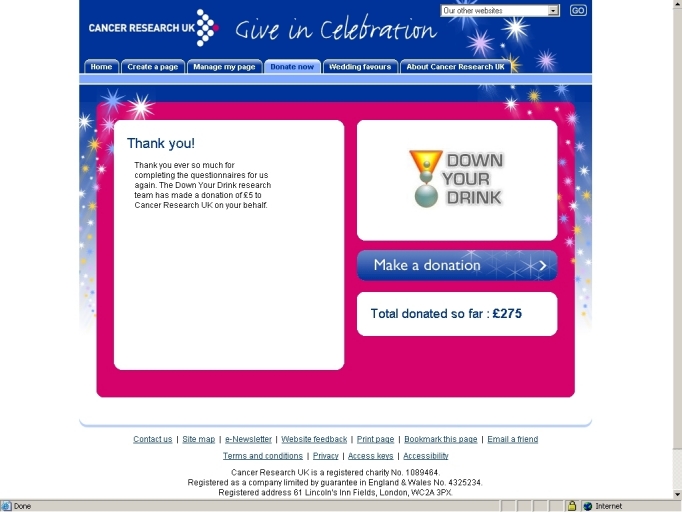
Screen shot of charity donation confirmation page

Participants responding in each incentive arm were sent an email (personally generated by author ZK), which thanked them for their time and contained, as appropriate a unique Amazon.co.uk voucher code and instructions on how to claim; a hyperlink to the charity’s website, which detailed the amount donated to Cancer Research UK as a result of participants completing the questionnaires (see Figure 2); and confirmation that they had been entered into a draw with a chance to win £250. Anonymity was maintained by sending the Amazon gift vouchers by email rather than requesting a postal address.

#### Study 2

**Figure 3:**
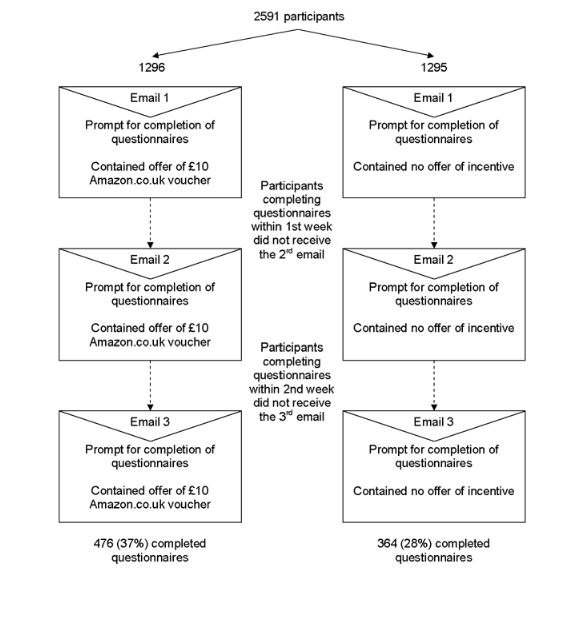
CONSORT flowchart: study 2

The results of study 1 informed the decision on level and type of incentive in study 2. In study 2, all DYD-RCT participants were randomized to receive either an offer of an incentive (£10 Amazon.co.uk voucher) or no incentive at the first request for data at the final (12-month) follow-up (between November 26, 2008, and September 9, 2009) (Figure 3). All participants received up to 3 email reminders with requests for provision of follow-up data. Each reminder contained a hyperlink to the study questionnaires, stressed the importance of providing follow-up data, and expressed our gratitude to participants. In addition, participants in the incentive arm were informed they would be sent a £10 Amazon.co.uk voucher on receipt of their completed study questionnaires. A further email with a unique Amazon.co.uk voucher code was sent on completion of questionnaires.

In both studies, randomization was performed by a computer-generated randomization sequence that triggered automatic emails to participants. Hence, randomization could not be subverted by the study team, and allocation was thus fully concealed. Randomization was stratified by DYD experimental group (DYD intervention vs DYD comparator). The randomization function in Java was used to generate random assignment.

### Outcomes

In both studies, the outcome was the proportion of participants who responded, defined as completing the questionnaires within 40 days of the first email reminder after randomization. Additional data already obtained at entry into the DYD trial, including age, gender, baseline weekly alcohol consumption, and DYD experimental group (intervention or comparator) were used to explore possible variability in outcome.

Data gathered for the economic analysis included the costs of developing the database for each study, researcher time in sending personalized emails, and costs of the incentives themselves.

### Analyses

For both studies, the sample size was calculated to detect a 6% difference in response rates between incentive and no incentive arms with 90% power at 5% significance level. The response rate in the no incentive arm was assumed to be 11% for study 1 and (building on the results of study 1) 26% for study 2. This gave total sample size requirements of 1468 for study 1 and 2400 for study 2.

The primary analysis compared response rates between the no incentive arm and incentive arm (3 incentive arms combined for the first study). For study 1, secondary analyses explored the differences between incentive types. Statistical significance was calculated using chi-square tests.

Subgroup analyses were conducted for gender, age, and heavy drinking at baseline (> 35 units per week for women and > 50 units per week for men where 1 unit = 8 g ethanol). Interactions between these variables and allocation to incentive in affecting response rates were tested on a risk difference scale using the binreg command in STATA. The statistical analyses were undertaken by authors EK and IW in STATA version 10 (StataCorp LP, College Station, TX).

A simple economic analysis was conducted for both studies. It cost £822 to set up a database for the research for study 1 and £1180 for study 2. Identifying which participants completed the questionnaires and were, therefore, eligible to receive an incentive or not and sending emails to deliver the incentive took 10 minutes per 10 participants at a cost (including overheads) of £0.95 per minute. In practice, offering incentives would involve some but not all of these costs. For example, if all participants were offered an incentive, then some of the selection and computer programming time would be saved. As the purpose of the economic evaluation was to compare the additional costs of incentives compared with the control condition of no incentives, a reasonable estimate of the additional setup costs is 50% of the database costs plus an additional minute of researcher time per incentive offered. The final costs of the scheme are those of the incentive. The cost-effectiveness ratios were calculated as the additional cost per successful additional completed follow-up, that is, the total cost of offering incentives divided by the number of additional responses (see Table 3). The economic analyses were undertaken by authors CG and ZK.

## Results

### Study 1

A total of 1226 participants were randomized to receive no offer of an incentive (n = 611) or offer of an incentive (n = 615) (Figure 1). The characteristics of participants randomized to each study arm were similar (Table 1). There was no significant difference in response rates of follow-up questionnaires between participants who received an offer of incentive (175/615, 29%) compared with those who did not receive offer of an incentive (162/611, 27%) (difference 2%, 95% confidence interval (CI) –3% to 7%), nor was there any significant difference in response rates between the 3 experimental arms (Amazon.co.uk voucher = 32%, charity donation = 27%, prize draw = 26%; *P* = .37) (Table 2). There were no significant interactions with gender, age, or heavy drinking at baseline (results not shown).

The costs associated with offering incentives in study 1 are outlined in Table 3. The incremental cost per successful follow-up in the incentive arm was £110 (£1432 total cost per 13 additional responses).

### Study 2

A total of 2591 participants were randomized to receive no offer of an incentive (n = 1295) or offer of a £10 Amazon.co.uk voucher (n = 1296) (Figure 3). Characteristics of participants randomized to each study group were similar (Table 1). There was a 37% (476/1296) response rate among those participants that received an offer of a £10 Amazon.co.uk voucher compared with a 28% (364/1295) response rate among those who did not receive an offer of an incentive (difference 9%, 95% CI 5% to 12%, *P* < .001) (Table 2). There were no significant interactions with the 3 baseline variables considered.

The incremental cost per successful follow-up in the incentive arm was £52 (£5802 total cost per 112 additional responses) (Table 3).

**Table 1:** Baseline characteristics of participants in study 1 and study 2

		Incentive	No incentive
**Female, %**			
	Study 1	54	54
	Study 2	58	59
**Age (years),** Mean (SD)			
	Study 1	37 (11)	37 (11)
	Study 2	38 (11)	38 (11)
**Baseline drinking (UK units),** Mean (SD)			
	Study 1	56 (37)	59 (42)
	Study 2	59 (37)	57 (42)
**DYD intervention arm, %**			
	Study 1	51	51
	Study 2	50	50

**Table 2:** Response rates for incentive groups in study 1 and study 2

Incentive Group		Total Randomized	Number of Responses	Response Rate	Difference	95% Confidence Interval
**Study 1**						
	Incentives (collectively)	615	175	29%	2%	–3% to 7%
	No incentive	611	162	27%		
	£5 Amazon voucher	206	66	32%		
	£5 charity donation	204	55	27%		
	£250 prize draw	205	54	26%		
**Study 2**						
	£10 Amazon voucher	1296	476	37%	9%	5% to 12%
	No incentive	1295	364	28%		

**Table 3:** Costs associated with offering incentives in study 1 and study 2

			Cost Per Person	Total Cost
**Study 1**				
	Setting up database		£0.67 per person	£411 (50% of total cost)
	Time sending confirmatory incentive email (per response to questionnaires)		£0.95 per person	£166
	**Incentive**			
		Amazon voucher	£5 (x66)	£330
		Charity donation	£5 (x55)	£275
		Prize draw	£250	£250
	Total			£1432
	Cost per extra follow-up response			£110
	Total cost per additional responses			£1432 per 13
**Study 2**				
	Setting up database		£0.46 per person	£590 (50% of total cost)
	Time sending confirmatory incentive email (per response to questionnaires)		£0.95 per person	£452
	Incentive (Amazon voucher)		£10 (x476)	£4760
	Total			£5802
	Cost per extra follow-up response			£52
	Total cost per additional responses			£5802 per 112

## Discussion 

These trials provide a valuable contribution to the limited literature on the use of incentives for reducing attrition in online trials. Study 1 found that promising a low level incentive (£5 Amazon.co.uk voucher, £5 charity donation, or prize draw for £250) had no significant impact on follow-up rates, whereas in study 2, a higher-level incentive (£10 Amazon.co.uk voucher) improved response rates by 9%. It should be borne in mind, however, that direct comparisons between the 2 studies are limited by differences in the study populations (those not initially responding in study 1 versus all respondents in study 2) and follow-up study time frames (3 and 12 months respectively). Notwithstanding these caveats, the higher incentive was also more cost-effective, in terms of costs per additional response. Researchers should, therefore, not assume that any level of incentive will necessarily improve follow-up rates.

The types of incentives offered in study 1 were comparable to those shown to have a positive impact on improving response rates to postal and electronic surveys [7]. However, collectively the incentives used in study 1 did not improve follow-up. In a trial of a health promotion website, the highest rate of retention was achieved with the highest value of incentive (ie, US $20 or £13) [10]. The findings of study 2 mirror this result. The survey literature suggests that unconditional incentives may be more effective than those conditional on completion of measures [7,9,25]. Our decision to promise an incentive on completion of the questionnaires, rather than unconditionally, was done for 2 reasons. The first was financial: online trials have the potential to recruit large numbers of participants (the DYD trial recruited 7935 people). If incentives were provided unconditionally to the entire sample, there would be substantial cost implications, and without the evidence to support this decision, the expense could not be justified. The second reason was methodological: providing unconditional incentives from the outset of the DYD pilot study might have encouraged multiple registrations for trial entry. In an online trial, with no face-to-face contact with trial participants, re-registration is a relevant concern [11].

Altruism is a commonly cited motive for trial participation [26-28], where participants take part in research for the benefit of others regardless of any benefit for themselves. There is a concern that the use of incentives may undermine altruistic reasons for participation. There is some evidence that altruistic motives are often accompanied by self-interest (conditional altruism), where participants are happy to help others if there is also some benefit for them in taking part in the trial [29-31]. These motivations have not surprisingly also been reported in the limited literature on trial retention, where participants are thought to remain in trials for personal benefit (ie, access to better treatment) as well as commitment to the trial and to help others [32,33]. Altruism is unlikely to have played a major role in the DYD-RCT, where participants were recruited while seeking feedback on, or help to, reduce drinking. Participants were not incentivized to take part in the DYD trial and had consented to complete follow-up questionnaires at study entry. Incentives were offered at follow-up as a “token of appreciation” for completing the questionnaires. Further research is needed to determine motives for entering and remaining in online trials and how this may impact on the use of incentives. Also warranting further exploration is the impact of socioeconomic status on the effectiveness of incentives, possible cultural differences in receptivity, and the underlying reasons for attrition, particularly related to the Internet setting (eg, Over the Internet, is it harder to establish rapport between participant and researcher and to obtain commitment on the part of the participant? What proportion of email reminders are caught in spam filters?).

Our conclusions are strengthened by the large sample sizes employed, the randomized design, and the completeness of the data. We were also able to inform the design of the second study using the results from the first. The £5 Amazon voucher in study 1 resulted in the highest response rate of the three incentive types, although not higher to a statistically significant degree (test results not reported). So, in the second study, participants were randomized to a higher-level incentive (£10 Amazon voucher). Study 2 was undertaken in a population and setting that were similar to study 1, the main differences being that study 1 was conducted among pilot DYD trial participants at 3 months who had not responded 1 week after an email request for follow-up, whereas study 2 was conducted among all main DYD trial participants eligible for 12-month follow-up within a defined time period. Our novel context of online trials is important, since it is likely to be the vehicle for an increasing number of studies of delivering health care and health promotion in the future. A potential limitation of the first incentive study is that it failed to meet its planned sample size because the DYD pilot phase ended slightly earlier than anticipated (due to programming commitments necessary for the commencement of the main DYD-RCT). For this reason and because response rates in the control arm were higher than expected, the results of study 1 were somewhat inconclusive, with a confidence interval including both no difference and the 6% difference in response specified in the power calculation.

This pair of studies has two important implications for researchers. Firstly, researchers should not assume that all levels of incentive would improve follow-up rates; instead, use of incentives for this purpose needs careful consideration and piloting of both level and type of incentive to be offered in a particular study population and setting. The second is that the costs of offering incentives can be substantial, and whether such costs are a good use of research funds needs to be considered. Further research that explores levels of different incentive types, offered and selected in different ways, and other means of reducing attrition in online trials should be prioritized if online health care delivery is to be well informed by strong research evidence.

## Abbreviations

APQ: Alcohol Problems Questionnaire
AUDIT: Alcohol Use Disorders Identification Test
CONSORT: Consolidated Standards of Reporting Trials
CORE: Clinical Outcomes for Routine Evaluation
DYD-RCT: Down Your Drink randomized controlled trial
LDQ: Leeds Dependence Questionnaire
TOT-AL: total past week alcohol consumption
